# Influence of Cold Atmospheric Plasma on Surface Characteristics and Bond Strength of a Resin Nanoceramic

**DOI:** 10.3390/ma16010044

**Published:** 2022-12-21

**Authors:** Xiaoming Zhu, Jiamin Shi, Xinyi Ye, Xinrong Ma, Miao Zheng, Yang Yang, Jianguo Tan

**Affiliations:** 1 Second Clinical Division, Peking University School and Hospital of Stomatology, Beijing 100101, China; 2 National Center of Stomatology, National Clinical Research Center for Oral Diseases, National Engineering Laboratory for Digital and Material Technology of Stomatology, Beijing Key Laboratory of Digital Stomatology, Beijing 100081, China; 3 Department of Prosthodontics, Peking University School and Hospital of Stomatology, Beijing 100081, China; 4 Guanghua School of Stomatology, Sun Yat-Sen University, Guangzhou 510055, China; 5 Department of Stomotology, Peking University Third Hospital, Beijing 100191, China

**Keywords:** resin nanoceramic, shear bond strength, cold atmospheric plasma, surface wettability

## Abstract

The purpose of this study was to investigate the effect of cold atmospheric plasma (CAP) treatment on resin nanoceramic (RNC) surface state and its bond strength with resin cement. RNC with different surface treatments were prepared: control, sandblasting treatment (SB), hydrofluoric acid etching (HF) and plasma treatment of helium gas (CAP-He) and argon gas (CAP-Ar). The prepared samples were measured by SEM, Ra, Rz, contact angle goniometer, and XPS for surface characteristics. The shear bond test of RNC was examined in nine groups: SB + saline coupling agent (SL), HF + SL, CAP-He/Ar, CAP-He/Ar + SL, SB + CAP-He/Ar + SL, and control. The bond strength between RNC and resin cement was compared using shear bond strength test, before and after thermocycling. After CAP irradiation, the surface topography maintained, while the surface water contact angle was significantly reduced to 10.18° ± 1.36° (CAP-He) and 7.58° ± 1.79° (CAP-Ar). The removal of carbon contamination and inducing of oxygen radicals was detected after CAP treatment. The bond strength was improved by CAP treatment, but varied on CAP gas species and combination methods. CAP of Ar gas had better SBS than He gas. After thermocycling, CAP-Ar + SL showed the maximized shear bond strength (32.38 ± 1.42 MPa), even higher than SB + SL group (30.08 ± 2.80 MPa, *p* < 0.05). In conclusion, CAP treatment of helium and argon can improve the bonding properties of RNC by improving surface wettability, and CAP of argon gas combined with silane coupling agent shows the highest bond strength.

## 1. Introduction

Resin-matrix ceramic is a new class of dental esthetic restorative material, consisting of a resin matrix filled with ceramic particles [1,2]. It has similar compositions and mechanical properties of both resin and ceramic, as “ceramic-like materials” [3,4]. In recent years, CAD/CAM resin-matrix ceramic materials have been developed rapidly, and widely used in indirect prosthesis, such as inlays, onlays and crowns, with easier and faster manufacturing [5,6]. However, for the long-term success of CAD/CAM restorations, great bonding between the restorative material and resin cement are required. Concerns have been raised regarding debonding and fracture rate of resin-matrix ceramic restorations, which may weaken their clinical performance [7,8].

According to different composition and microstructure, CAD/CAM resin-matrix ceramic materials are mainly divided into two types: polymer-infiltrated hybrid ceramic (PIHC) and resin nanoceramic (RNC) [2,3]. For PIHC, it’s typically composed of a dual network: a feldspathic ceramic network and a poly network [3]. It has been demonstrated great bonding properties after etching with hydrofluoric acid, similar as feldspathic ceramics [8,9]. While for RNC, it consists of a highly cured resin matrix reinforced with silica or zirconia nanoparticles. Since RNC is non-ecthable material, sandblasting combined with silane coupling agent has been recommended before its bonding [10,11,12]. However, sandblasting may damage the structure of resin matrix, decreasing its flexural strength, even causing fracture [13]. Is there an effective surface treatment method for improving the bonding performance of RNC without damaging its mechanical strength?

Cold atmospheric plasma (CAP), as an efficient and clean surface treatment method, has been used to improve the bonding properties of other all-ceramic dental materials, like zirconia and glass-matrix ceramics [14,15,16,17]. It can effectively modify the physicochemical properties of those materials, by increasing their hydrophilicity [18,19]. The bond strength of RNC can also be enhanced by increasing its surface energy, wettability and reactivity [20,21]. Whether CAP treatment an effective method of RNC bonding? More supported evidence is still needed.

The purpose of this study was to evaluate the effect and mechanism of CAP on resin bonding to RNC, comparing with sandblasting and hydrofluoric acid etching. Physical and chemical alterations of RNC after treatment were assessed, as well as the shear bond strength (SBS) before and after thermocycling. The first null hypothesis was that CAP treatment did not change the surface characteristics of RNC and improve the bond strength of RNC. Besides, CAP of different kinds of gas, like helium or argon, and combinations with sandblasting were also evaluated in this study. The second null hypothesis was that there’s no difference between various CAP methods. This study was expected to prove CAP as a new method improving RNC’s bonding properties. Besides, the mechanism of CAP demonstrated in this study may broaden its application in dental materials’ modification.

## 2. Materials and Methods

### 2.1. Preparation of RNC Specimens

**Figure 1:**
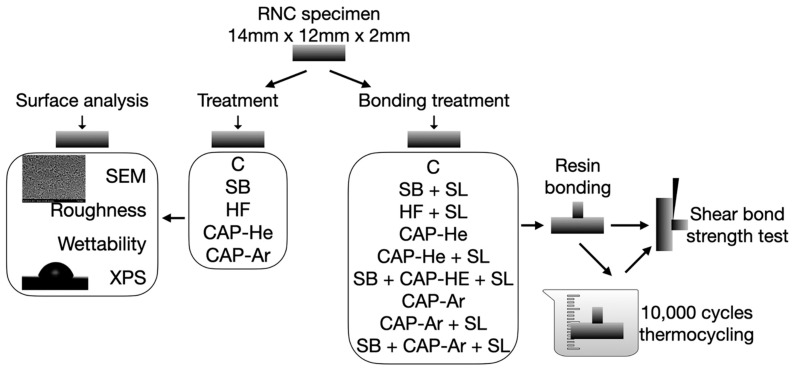
The schematic design of the study.

The RNC used in this study was Renci CAD/CAM resin nano ceramic (UPCERA, Shenzhen, China) widely used in China in recent years [1]. The main composition of RNC and other materials used in this study was listed in Table 1. The RNC blocks were sectioned into square specimens (14 mm × 12 mm × 2 mm), using a slow-speed diamond wafering blade (Isomet 1000 Precision Saw, Buehler; Lake Bluff, IL, USA). They were then wet ground (Automet 500; Buehler, Esslingen, Germany) by 600-grit SiC for about 1 min. Before further experiments, all specimens were ultrasonically cleaned and thoroughly dried. The schematic design of the study was shown in Figure 1.

### 2.2. Surface Treatment and Analysis

All RNC specimens were submitted into five groups for treatment:

- Control group (C): specimen with no treatment.
- Sandblasting group (SB): 50 μm Al_2_O_3_ particles (COBRA, Rengert, Germany) were sandblasted for 20 s at a pressure of 0.1 MPa and a distance of 10 mm. After sand blasting, ultrasonically cleaning (using deionized water for 5 min) and drying was carried out for the specimens.
- Hydrofluoric acid etching group (HF): A hydrofluoric acid agent (IPS Ceramic Etching Gel, Ivoclar Vivadent, Schaan, Liechtenstein) was applied for 60 s with a disposable brush, rinsed with deionized water for 1 min and then thoroughly dried.
- CAP jet with helium gas group (CAP-He): specimen was treated with a CAP jet with a helium flow rate for 120 s, at a distance of 10 mm.
- CAP jet with argon gas group (CAP-Ar): The surface was treated with a CAP jet with an argon flow rate for 120 s, at a distance of 10 mm.

**Figure 2:**
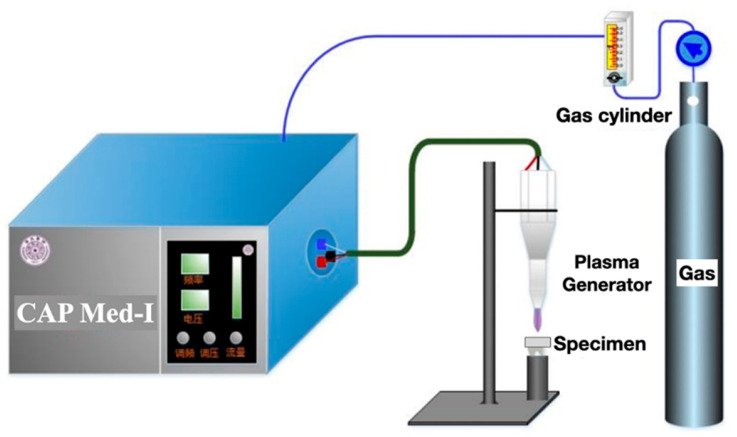
CAP-Med I equipment used in this study for plasma jet generation.

In both CAP groups, the plasma was produced by CAP Med-I (Figure 2) at the condition of 2.8 kV and 17 kHz, with the gas flow rate of 8.1 slpm. The other details of this equipment have been reported before [22,23,24].

After treatment, specimens in all five groups (five specimens per group) were first observed in a scanning electron microscopy (SEM) (S-4800, Hitachi, Japan) at 35 mA for 85 s, after sputter-coated with Au-Pd alloy. The surface roughness μm) of specimen was measured by a stylus surface profilometer (SJ-401, Mitutoyo, Japan), including Ra (arithmetical mean height, in μm) and Rz (maximum height of surface roughness profile, in μm). They were both determined with a cut-off value of 0.8 mm, measurement length of 4 mm. For each specimen, three times was measured at different areas and five specimens per group.

The wettability of surface to water was determined by a contact angle goniometer (SL200, USA Kino Industry, Norcross, GA, USA). Static contact angle was measured using tangential line method. On each specimen, 1 μL deionized water droplet was applied with an automatic piston syringe. Three randomly selected points on each specimen were examined, and five specimens per group.

Electron spectroscopy for chemical analysis (ESCA) was used to evaluate the composition of the treated surface. It was conducted by X-ray photoelectron spectroscopy (Kratos Analytical, Manchester, UK) to evaluate the intensity of carbon, oxygen and silicon on the surface. The binding energy of each spectrum was calibrated with C1s (284.8 eV).

### 2.3. Shear Bond Strength Test

180 prepared RNC specimens were randomly divided into nine groups according to different surface treatments (Table 2).

A light-cured resin material (Clearfil AP-X, Kuraray, Tokyo, Japan) was fabricated intio resin cylinders (4 mm in diameter and 4 mm in height). The process has been reported in our previous study [17]. After prepared, the resin cylinder was bonded onto group-treated RNC’s surface, using self-adhesion resin cement (RelyX U200 3M ESPE, St. Paul, MN, USA) at a static load of 5 N. 40 s of LED light irradiation was applied, before cement excess was carefully removed.

After bonding, half of samples in each group were submitted to the shear bond strength test immediately (n = 10), and the other half were submitted to a thermocycling aging for 10,000 cycles (5 °C~55 °C), before shear bond strength test (n = 10).

The shear fracture loads were measure by a universal mechanical testing machine (EZ-L, SHIMADZU, Japan). The crosshead had a speed of 1 mm/min until failure. The SBS was calculated as:

SBS (MPa) = Maximum load,F (N)/ Bonding Area,S (mm^2^)

The failure mode after shearing was observed (SMZ-10, Nikon, Japan) and classified as: adhesive failures, mixed failures and cohesive failures.

### 2.4. Statistical Analysis

All the data were collected and expressed as means and standard deviations, after normal distribution test. They were tested for statistical significance using one-way ANOVA analysis of variance, with a significance level of 0.05. Post-hoc analysis using the Tukey method was performed to detect pairs of groups with statistically significant differences. The data were statistically analyzed using SPSS software version 25.0 (SPSS, IBM Corp., Chicago, IL, USA).

## 3. Results

### 3.1. Surface Analysis of RNC after Treatment

**Figure 3:**
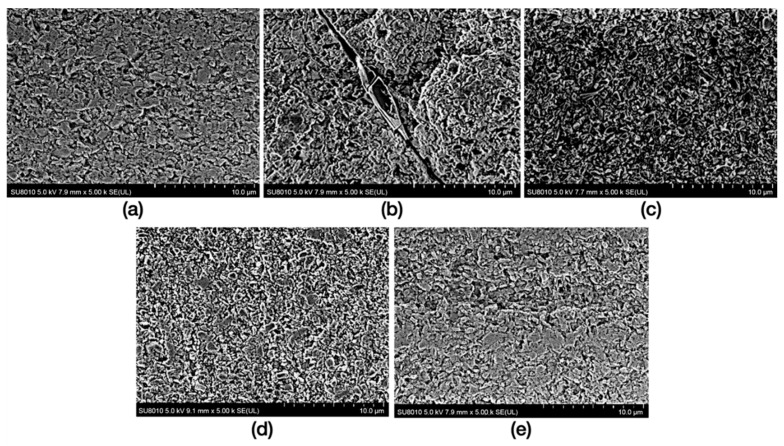
Scanning electron microscopy images of RNC specimens. (**a**) specimen in control group; (**b**) specimen in SB treatment; (**c**) specimen in HF treatment; (**d**) specimen in CAP-He treatment; (**e**) specimen in CAP-Ar treatment.

The surface morphology of RNC in different groups was shown in Figure 3. Compared to control group, SB presented a roughed surface with several grooves and faceted slits, embedded with few particles. While HF produced a slightly porous morphology, with glassy matrix removal. Both CAP-He and CAP-Ar did not result in visible change to RNC surface.

Surface roughness of RNC specimen was presented in Table 3. The Ra value of un-treated RNC was 0.09 ± 0.01 μm. There’s no significant difference after CAP-He and CAP-Ar treated (0.09 ± 0.02 μm for CAP-He, and 0.10 ± 0.02 μm for CAP-Ar, *p* > 0.05). After SB and HF treatment, both Ra values increased (0.47 ± 0.05 μm and 0.17 ± 0.02 μm respectively, *p* < 0.05). The Rz value of CAP-Ar (0.79 ± 0.14 μm) was slightly larger than CAP-He (0.75 ± 0.13 μm), but with no significant difference (*p* = 0.99). The Rz values of other groups showed the same trend.

**Figure 4:**
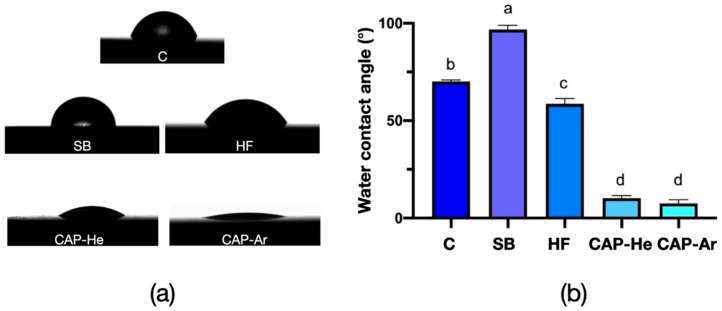
Water contact angle of RNC specimens. (**a**) classical images of each group; (**b**) statistical analysis of water contact angle values, and different letters denote significant differences (*p* < 0.05).

The water contact angle measurements were presented in Figure 4. Compared to the high value of un-treated RNC (70.05° ± 0.86°), HF decreased the water contact angle into 58.64° ± 2.72° (*p* < 0.05), while SB increased the value (96.21° ± 2.03°, *p* < 0.05). While, for both CAP groups, the water contact angles decreased significantly (*p* < 0.01), 10.18° ± 1.36° for CAP-He, and 7.58° ± 1.79° for CAP-Ar, showing the greatly enhanced hydrophilicity.

**Figure 5:**
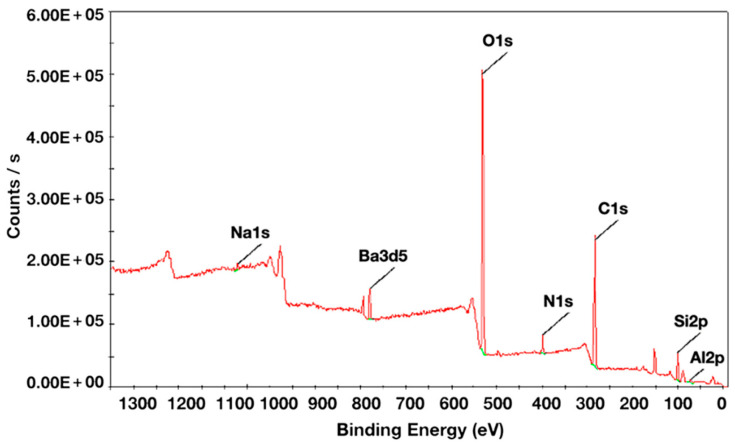
XPS wide-scan spectra of RNC specimen.

The XPS analysis of RNC specimens showed peaks of C 1s, O 1s, N 1s, Ba 3d5, Si 2p (Figure 5). Table 4 showed the main chemical compositions (C 1s, O 1s, Si 2p) and C/O ratio of each group. After the CAP-He and CAP-Ar treatment, the content of C1s decreased significantly from 51.17% to 29.12% and 28.14%, while the oxygen content increased from 36.90% to 50.93% and 51.2%. C/O ratio was 0.57 for CAP-He, and 0.55 for CAP-Ar. Besides, the silicon content decreased after hydrofluoric acid.

### 3.2. Shear Bond Strength of RNC

As listed in Table 5, the immediate shear bond strengths of RNC in all treatment groups were significantly higher than control group (16.83 ± 1.77 MPa, *p* < 0.05). Groups SB + SL, SB + CAP-He + SL, CAP-Ar, CAP-Ar + SL and SB + CAP-Ar + SL all presented the comparably SBS, presenting the highest values among all treated groups (*p* < 0.05). HF + SL presented the lowest SBSs (21.17 ± 1.37, *p* < 0.05) among all treatment groups.

After 10,000 thermocycling, the binding area of samples maintained the same, while SBS values of treated groups were all significantly higher than control (13.38 ± 3.90 MPa, *p* < 0.05). Group CAP-Ar + SL (32.38 ± 1.42 MPa) exhibited the highest SBSs (*p* < 0.05) among all treatment groups, while groups SB + SL, SB + CAP-He + SL, CAP-Ar and SB + CAP-Ar + SL presented the comparable SBS (*p* > 0.05). Compared with immediate shear bond strength, the SBS values in most groups decreased significantly after thermocycling (*p* < 0.05). While only in SB + SL and CAP-Ar + SL, SBSs after aging maintained the comparable values (*p* > 0.05) compared with their immediate SBSs.

**Figure 6:**
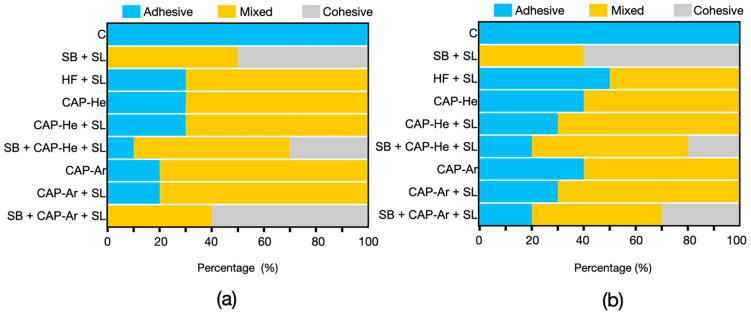
Failure mode distributions of shear bond test before (**a**) and after (**b**) thermocycling.

Due to the mechanical properties of RNC, the failure modes were classified as: adhesive failure (fractured at the RNC/resin cement bonding interface), mixed failure (fractured occurred at both bonding interface and inside resin cement) and cohesive failure (fractured inside RNC specimens or resin cement). The failure modes (Figure 6) in control group were all adhesive failures. In SB + SL group, before and after thermocycling, the failure mode showed large proportion of cohesive fractures predominantly within the RNC specimens. There’re also cohesive fractures in groups SB + CAP-He + SL and SB + CAP-Ar + SL. In other treated groups, there were only mixed and adhesive failures, and the percentage of adhesive failures increased after thermocycling.

## 4. Discussion

Great and endurable bonding with resin cement is prominent for dental hybrid and ceramic materials. As a novel CAD/CAM material, RNC has displayed great clinical performance with relatively high bonding strength [2,3,4,5,6]. The present study evaluated the effect of helium and argon CAP treatment on surface characteristics and bond strength of RNC. According to the results, the first null hypothesis stating that CAP treatment dos not change RNC’s surface characteristics and bond strength should be rejected. Moreover, the second null hypothesis of the study stating that there’s no difference between helium or argon CAP or combinations of CAP treatment should also be rejected.

For RNC, consisting of a highly cured resin matrix reinforced with silica nanoparticles, controlled sandblasting and silane coupling agent was recommended before bonding rather than acid [11,12,25]. After sandblasting, the roughened surface may allow resin cement to flow into these micro-retentions, and form a stronger micro-mechanical interlock. However, it has been reported that sandblasting could initiate surface defects that may compromise the mechanical properties of resin nanoceramic materials [13]. Tekçe et al. [25] also demonstrated that excessive sandblasting (50 µm alumina 0.2 MPa for 30 s) produced large crack propagating along the material, and also decreased the microtensile test values after 5000 thermocycling. The residual sandblasting particles may also affect its bonding properties. Ultrasonic cleaning was necessary after sandblasting for unless 5 min [10]. In this present study, the mild sandblasting (50 μm alumina at 0.1 MPa for 20 s) was used, according to the references [10,11,12,13,25] and our pre-test. The SEM images presented a roughed surface with several grooves and faceted slits embedded with few particles. Combining with silane coupling agent, it improved the shear bond strength more than hydrofluoric acid, and there’s no significant difference between immediate and after thermocycling. But the failure modes of specimens in SB + SL group exhibited a large proportion of cohesive failures. Cohesive fractures predominantly within the RNC revealed that the shear resistance of RNC itself, weakened by sandblasting, was lower than the bonding strength with resin cement. Nevertheless, group SB + SL, with mild sandblasting, was still designed as the gold standard in our shear bond strength test to compare the other pre-bonding methods.

Hydrofluoric acid etching, by dissolving the glassy matrix containing silica (SiO_2_), also increases the surface roughness of ceramic materials [9]. It can improve the micro-mechanical retention of the applied primer, and also enhance surface’s wettability, promoting an optimal bond strength. According to the results, after HF acid etching for 60 s, RNC surface became porous and XPS presented significantly decreased Si content from 9.75% to 0.09%. However, in shear bond test, HF + SL led to a relatively low SBS value. This may be related to the chemical structure of RNC, less feldspar ceramic phase and much more polymeric phase, which is less susceptible to hydrofluoric acid than PHIC [3].

CAP is an artificial plasma created by partially ionized gas. It can increase surface energy and hydrophilicity, by generating highly reactive particles, such as ions, electrons and free radicals. It has been demonstrated an effective way to modify surface of dental ceramics and natural tooth for better hydrophilicity [23,26,27]. In this present study, CAP of He and Ar gas both enhanced RNC’s wettability significantly. CAP-Ar even modified RNC surface into super hydrophilicity (<10°), while the surface morphology maintained the same. The XPS analysis showed a significant decrease in the C% after He and Ar CAP treatment. The same phenomenon was reported by Henningsen et al. [28] that plasma was capable of decreasing carbon-rich contaminants such as C-OH, C = O, COOH radicals, which are known to compromise surface’s bonding strength. Furthermore, the CAP treatment on RNC surface produced an increase in the oxygen content. A high level of reactive -O radicals (i.e., hydroxyl free radicals, excited oxygen ions, and atomic oxygen) can be produced by plasma [17,22,29], thereby highly increasing materials’ hydrophilicity. The C/O ratio, representing surface wettability, was 0.57 for CAP-He and 0.55 for CAP-Ar, both much lower than control and other treated RNCs. It demonstrated that the collisions between RNC surface and reactive oxygen species, combining with the reduced carbon content, both contributed to the enhancing of hydrophilicity.

According to the hydrophobic recovery theory [30], when a plasma-treated surface is exposed to the atmosphere, the reactive specimens will react with elements and impurities in the atmosphere, and diminish over time [22,29]. Barquete et al. reported that the improved bonding properties was available only if the surface was cemented within 8 h after CAP irradiation [29]. So, in this study, the bonding process was immediately followed the CAP treatment. Since sandblasting was the gold standard and suggested by manufacture, CAP treatment was designed in this study as a substitute for sandblasting. This present study was mainly focused on whether CAP was a comparable or even better method than sandblasting. The application of CAP has three modes for each gas: CAP alone, CAP combined with silane coupling agent, and CAP combined with SB + SL (CAP treatment after sandblasting, and then silane coupling agent).

Although the wettability of the RNC surface was maximized when plasma irradiation was performed using both helium and argon gas, the SBS values of CAP-He and CAP-Ar were not both maximized. After CAP-He treatment, the bonding strength of RNC has been improved a little, only SB + CAP-He + SL achieved the equal SBS value to SB + SL. While after thermocycling, all those application modes had lower SBSs than gold standard (SB + SL). Fortunately, CAP-Ar had better results. All groups with CAP-Ar had comparably maximized SBSs. Besides, after thermocycling, CAP + SL produced the highest SBS value among all groups. This positive result of may be related to the micro-etching effect and a large amount of oxygen particles, especially -OH radicals, produced by CAP of argon [31,32,33]. During the discharge with high intensity, water in the air may be ionized into -OH radicals, promoting super-hydrophilic surface [33]. Furthermore, argon plasma can also promote polymerization initiation of resin-based materials [34], promoting better properties and bonding performance of dental composite restorations. To illustrate the different mechanism of argon and helium CAP on RNC, further investigation like Atomic Force Microscope (AFM) or Raman spectra may be needed. Different reactive particles or radicals should be further examined. Nevertheless, it has been demonstrated that micromechanical roughening could increase CAD/CAM hybrid materials’ bond strength, more than other chemical modifications, especially after thermocycling aging [35,36]. The study of Castro EF et al. [37] also presented a negative result that no significant benefit was found in RNC’s bonding, using plasma alone or combined with a bonding agent. The possible reason might be different CAP devices and conditions. In their study, plasma was generated through a hand-held unit at a flow rate of 1 slpm, and was applied for only 30 s. While in this present study, the intensity of plasma was much greater with a flow rate of 8.1 slpm, and was applied for 120 s. Besides, the RNC used in this study contains 55–85 wt% glass ceramic, including 0.1 μm~1 μm B_2_O_3_ and 5 nm~50 nm SiO_2_. It has been widely used in China. While there’s another widely-used RNC, the Lava Ultimate (3M ESPE, Seefeld, Germany), which contains SiO_2_, ZrO_2_ and aggregated ZrO_2_/SiO_2_ cluster. Different ceramic filler particles in these RNC materials may also lead to differential reaction to CAP treatment. Besides, Ahn JJ et al. reported a greater bond strength of zirconia after CAP treatment combined with sandblasting [38]. While in this study, for RNC, CAP treatment combined with sandblasting did not promote better bond strength. It may be related to the high content of crystalline in zirconia. The air-abrasion can produce a roughed surface, providing more opportunities for CAP reaction and a larger bonding area, without weakening zirconia’s structure. But for RNC, as already said, sandblasting could initiate damage of structure that may compromise the mechanical strength and bonding properties [13,25]. In this study, after thermocycling, CAP-Ar combined with SB + SL had lower SBS values and more cohesive failures than CAP-AR + SL.

Although the present study showed some interesting and meaningful aspects regarding the influence of helium and argon CAP treatment on the bonding performance of RNC. The limitations should be noted that only one kind of RNC materials was used in this study, and only self-adhesive resin cement was tested. The other resin cements, multi-functional primer agent may also affect bonding properties of RNC. Besides, the thermocycling in vitro cannot imitate the real aging performance in clinical situations. Although it has been reported that approximately 10,000 thermo cycles related to 1 year life in vivo [39]. The simulation of mastication forces and saliva both can influence the long-term success of bond, which should be addressed in future investigations.

## 5. Conclusions

The effectiveness of cold atmospheric plasma irradiation for RNC surface treatment was demonstrated. Plasma treatment with helium and argon gas do not change the surface morphology of RNC, but can significantly improve the surface wettability of this material, by removing carbon contamination and introducing active oxygen radicals. Different CAP treatments resulted in differential bonding properties, and the argon CAP combined with silane coupling agent improved the highest bonding strength. It was suggested that CAP could be a new surface treatment method for RNC bonding.

## Figures and Tables

**Table 1:** Materials used in this study with their manufactures and main compositions.

Materials	Manufacture	Main Composition *
resin nanoceramic	Renci Upcera	13–43 wt% polymer, 55–85 wt% glass ceramic (including 0.1 μm~1 μm B_2_O_3_, 5 nm~50 nm SiO_2_)
hydrofluoric acid	IPS ceramic etching gel Ivoclar Vivadent	≤ 5% hydrofluoric acid
silane coupling agent	RelyX ceramic primer 3M ESPE	Ethanol, 3-trimethoxysilylpropyl methacrylate
composite resin	Clearfil AP-X Kuraray	Base resin: Bis-GMA, TEGDMA Filler: 85 wt% silanated barium glass filler of irregular shape (700 nm), and silanated silica filler (100–1500 nm)
resin cement	RelyX U200 3M ESPE	Bi-functional (meth) acrylate; Inorganic fillers (43% by volume)
Bis-GMA: Bisphenol A glycerolate dimethacrylate; TEGDMA: Triethylene glycol dimethacrylate;		

* The information was provided by the manufacturers.

**Table 2:** Different surface treatment of nine groups before binding.

Groups	Surface Treatment before Bonding
control	no treatment
SB + SL	sandblasting + silane coupling agent *
HF + SL	hydrofluoric acid + silane coupling agent
CAP-He	CAP jet with helium gas
CAP-He + SL	CAP jet with helium gas + silane coupling agent
SB + CAP-He + SL	sandblasting + CAP jet with helium gas + silane coupling agent
CAP-Ar	CAP jet with argon gas
CAP-Ar + SL	CAP jet with argon gas + silane coupling agent
SB + CAP-Ar + SL	sandblasting + CAP jet with argon gas + silane coupling agent

* RelyX ceramic primer (3M ESPE, St. Paul, MN, USA) as silane coupling agent was applied to RNC specimens for 1 min, and dried with oil-free air spray.

**Table 3:** Surface roughness of RNC specimens.

Groups	Ra (Mean ± SD, μm)	Rz (Mean ± SD, μm)
control	0.09 ± 0.01 ^c^	0.71 ± 0.12 ^C^
SB	0.47 ± 0.05 ^a^	3.87 ± 0.33 ^A^
HF	0.17 ± 0.02 ^b^	1.38 ± 0.13 ^B^
CAP-He	0.09 ± 0.02 ^c^	0.75 ± 0.13 ^C^
CAP-Ar	0.10 ± 0.02 ^c^	0.79 ± 0.14 ^C^

Ra: arithmetical mean height, Rz: maximum height of surface roughness profile. Different letters in columns showed significant differences (*p* < 0.05) between groups.

**Table 4:** Main chemical compositions on RNC surfaces.

Groups	Atomic %			C/O
	C 1s	O 1s	Si 2p	
C	51.17	36.90	9.75	1.39
SB	56.30	33.94	7.69	1.66
HF	69.68	27.50	0.09	2.53
CAP-He	29.12	50.93	16.62	0.57
CAP-Ar	28.14	51.2	17.82	0.55

**Table 5:** Shear bond strength (SBS) of RNC specimens in different groups.

Groups	SBSs (Mean ± SD, MPa)	
	Immediate (n = 10)	After Thermocycling (n = 10)
control	16.83 ± 1.77 ^d^	13.38 ± 3.90 ^F^*
SB + SL	31.50 ± 2.74 ^a^	30.08 ± 2.80 ^B^
HF + SL	21.17 ± 1.37 ^c^	17.47 ± 2.04 ^E^*
CAP-He	27.93 ± 1.74 ^b^	23.02 ± 2.62 ^D^*
CAP-He + SL	28.51 ± 1.71 ^b^	25.98 ± 1.74 ^C^*
SB + CAP-He + SL	32.10 ± 2.57 ^a^	28.77 ± 3.07 ^B,C^*
CAP-Ar	33.97 ± 2.04 ^a^	28.28 ± 2.56 ^B,C^*
CAP-Ar + SL	33.78 ± 1.60 ^a^	32.38 ± 1.42 ^A^
SB + CAP-Ar + SL	32.52 ± 2.31 ^a^	28.90 ± 1.82 ^B,C^*

Different letters in columns showed significant differences (*p* < 0.05) between groups. * Represented a significant difference (*p* < 0.05) between immediate and after thermocycling SBSs.

## Data Availability

Not applicable.
